# Enhancing View Synthesis with Depth-Guided Neural Radiance Fields and Improved Depth Completion

**DOI:** 10.3390/s24061919

**Published:** 2024-03-16

**Authors:** Bojun Wang, Danhong Zhang, Yixin Su, Huajun Zhang

**Affiliations:** School of Automation, Wuhan University of Technology, Wuhan 430070, China; bojun.wang@whut.edu.cn (B.W.); suyixin@whut.edu.cn (Y.S.); zhanghj@whut.edu.cn (H.Z.)

**Keywords:** neural radiance fields, volume rendering, view synthesis, image-based rendering, depth priors, rendering accelerations

## Abstract

Neural radiance fields (NeRFs) leverage a neural representation to encode scenes, obtaining photorealistic rendering of novel views. However, NeRF has notable limitations. A significant drawback is that it does not capture surface geometry and only renders the object surface colors. Furthermore, the training of NeRF is exceedingly time-consuming. We propose Depth-NeRF as a solution to these issues. Specifically, our approach employs a fast depth completion algorithm to denoise and complete the depth maps generated by RGB-D cameras. These improved depth maps guide the sampling points of NeRF to be distributed closer to the scene’s surface, benefiting from dense depth information. Furthermore, we have optimized the network structure of NeRF and integrated depth information to constrain the optimization process, ensuring that the termination distribution of the ray is consistent with the scene’s geometry. Compared to NeRF, our method accelerates the training speed by 18%, and the rendered images achieve a higher PSNR than those obtained by mainstream methods. Additionally, there is a significant reduction in RMSE between the rendered scene depth and the ground truth depth, which indicates that our method can better capture the geometric information of the scene. With these improvements, we can train the NeRF model more efficiently and achieve more accurate rendering results.

## 1. Introduction

Novel View Synthesis (NVS) involves reconstructing a 3D representation from captured images for rendering from new viewpoints. Traditional methods like Image-Based Rendering (IBR) and Multi-View Stereo (MVS) have been widely used. However, the emergence of Neural Radiance Fields (NeRF), with its superior handling of complex scenarios such as occlusions, reflections, and transparency, signals a promising direction in NVS research. By modeling a scene as a continuous volumetric function parameterized by a multilayer perceptron (MLP), NeRF is capable of generating photorealistic renderings that exhibit detailed geometry and view-dependent effects.

NeRF, despite its remarkable achievements, has certain limitations. When the scene is captured from a limited number of sparsely-distributed viewpoints, NeRF’s ability to accurately represent intricate geometry and appearance may lead to the presence of several artifacts arising from an imperfect density distribution. The underlying reason is that NeRF relies solely on RGB values from input images to determine the relationships among input views. Therefore, obtaining high-quality rendering results requires feeding adequate images with diverse perspectives. Training NeRF is notoriously time-consuming, and may take up to 10–12 h to complete using an NVIDIA RTX 3060 on a regular room-sized scene, becoming a significant bottleneck to NeRF’s robust adoption. Moreover, NeRF runs the risk of overfitting when not provided with enough input views. In such instances, NeRF can only render the surface color of an object but is incapable of precisely capturing the depth information of the scene.

DoNeRF [1] introduces an oracle network to predict ideal sampling positions for the ray-tracing shading network, significantly reducing inference time. However, it does not substantially improve training speed, leaving the NeRF training duration issue unresolved. EfficientNeRF [2] proposes a different approach to the sampling strategy by advocating for effective and critical sampling at both coarse and fine stages. It also utilizes NeRFTree to efficiently represent 3D scenes, leading to faster caching and querying and improving rendering speed. Nonetheless, EfficientNeRF does not eliminate the problem of color and geometry mismatches resulting from overfitting.

**Figure 1:**
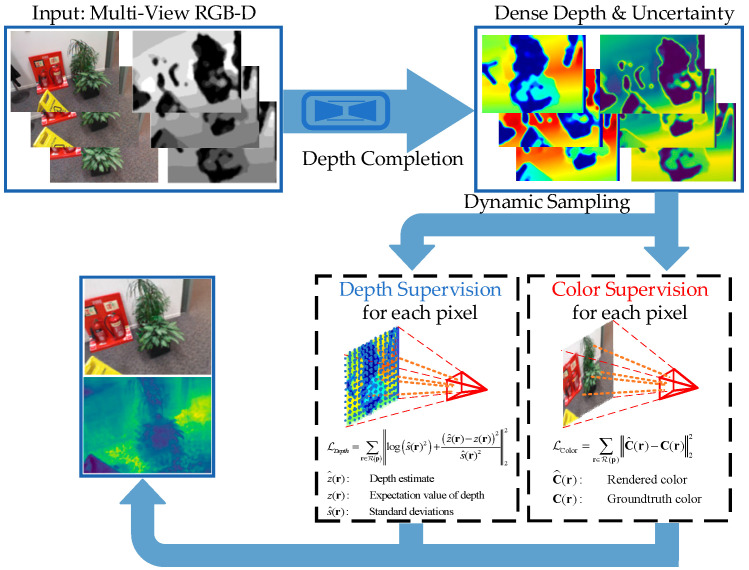
Method overview.

This study addresses the above-mentioned challenges by proposing a method to supervise NeRF using both color and depth information. Additionally, it introduces a novel sampling approach that effectively reduces training time without compromising rendering quality. Since the depth maps generated by consumer-grade RGB-D cameras often suffer from noise, missing data, and holes, we deploy a depth completion technique to generate dense and accurate depth maps for feeding into NeRF. Depth completion endeavors to densify sparse depth maps by predicting depth values on a per-pixel level. It holds a crucial position in several domains, including 3D reconstruction, autonomous driving, robot navigation and augmented reality. Deep learning-based approaches have emerged as the predominant solutions for this task, demonstrating notable achievements in recent advancements. Considering the substantial computational resources required by NeRF, the utilization of deep learning-based methods is not viable. This study employs the OpenCV-based depth completion algorithm, which is capable of running in real-time on a CPU with comparable accuracy to mainstream deep learning-based methods for depth completion. Furthermore, our approach to utilizing depth information is entirely different from the methods mentioned earlier. Rays are cast from the camera coordinate center towards these 3D points, theoretically terminating at these 3D points. We apply a depth loss to encourage the termination distribution of the rays to be consistent with the 3D points in space. Our method’s pipeline is shown as Figure 1.

In summary, this study introduces an RGB-D neural radiance field that incorporates color and depth measurements, employing an implicit occupancy representation. The introduction of depth information demonstrates a notable positive influence on the rendering quality, surpassing the performance achieved through training with RGB data alone. Moreover, we propose a novel dynamic sampling approach, which utilizes depth information for more precise sampling. By reducing unnecessary sampling points, the dynamic sampling technique significantly accelerates the training process and lowers the computational load compared to the original NeRF.

The main contributions of this study are as follows:

1. Introducing depth completion to NeRF to provide dense and accurate depth maps.
2. Enhancing the sampling efficiency by utilizing dense depth information.
3. Proposing a mechanism for generating depth rays in NeRF to enhance rendering quality, improve accuracy, and ensure consistent scene depth.

## 2. Related Work

Our proposed Depth-NeRF utilizes a series of RGB-D images as input and trains an MLP to acquire a comprehensive volumetric scene representation from the observed data. The depth information is utilized to supervise NeRF training and determine the distribution of sampling points in order to accurately capture the depth at which a ray terminates using fewer sampling points. In the following sections, we will discuss related work pertaining to this research.

### 2.1. Neural Volume Rendering

The well-known NeRF [3] has achieved impressive results using a simple approach. An MLP takes 3D points and view direction as input and generates density and color values as output. However, NeRF has some drawbacks, including long training and rendering times, the need for separate models for each scene, and its limitation to static scenes. The problem of static scenes is addressed in [4,5,6]. NeRF models have been extended and generalized in [7,8,9,10] by incorporating fully convolutional image features, utilizing generator-discriminator architectures, and leveraging meta-learning techniques. These advancements have enhanced their ability to handle a wider range of scenes and produce more realistic renderings. In [11], a CNN-based encoder and an MLP-based decoder are employed to compute density and color values for each point in space, enabling the creation of realistic and visually appealing images. In [12], data-driven methods are used to train a deep network for predicting volumetric representations and employ alpha-compositing to render photographs from novel viewpoints. In [13], the optimization of a hybrid approach that combines convolutional networks with scene-specific voxel grids is explored. This combination effectively addresses the discretization artifacts resulting from the use of low-resolution voxel grids. In [14], neural networks are employed to address gaps and enhance the overall quality of a textured geometric representation. BakedSDF [15] and MonoSDF [16] both utilize a signed distance function (SDF) to represent scene geometry. Neuralangelo [17] leverages the representation capabilities of 3D hash grids in combination with neural surface rendering.

### 2.2. Neural Radiance Field with Depth

Prior research has delved into the application of depth information in view synthesis and NeRF training. In the NerfingMVS [18], a monocular depth network is adapted to the specific scene by fine-tuning it on a sparse Structure from Motion (SfM) combined with Multi-view Stereo (MVS) reconstruction. The adjusted depth priors are subsequently utilized to steer the sampling procedure during volume rendering. The adapted depth priors are subsequently employed to guide and supervise the sampling process of volume rendering. Meanwhile, DS-NeRF [19] introduces improvements to NeRF by incorporating a direct supervision mechanism for its density function. This is accomplished through the utilization of a specific loss function that encourages the alignment of ray termination depth distribution with a given 3D keypoint. DONeRF [1] reduces inference costs by employing a depth oracle network that computes sample points’ locations in a single evaluation. This replaces NeRF’s MLP-based raymarching with a compact local sampling strategy, which prioritizes crucial samples near surfaces. The primary objective is to improve efficiency and prioritize relevant information during rendering. In addition, ref. [20] incorporates depth information within the Mip-NeRF [21] framework and models depth uncertainty to enhance geometry accuracy, reduce artifacts, and improve the efficiency of both training and prediction. The integration of depth information with NeRF for enhanced reconstruction, as presented in [22], involves the utilization of a truncated signed distance function (TSDF) instead of density. This approach incorporates two networks that have an impact on both the training and prediction time.

### 2.3. NeRF Training Acceleration

In [23,24,25], the issue of lengthy inference time in NeRF is addressed by employing a compact MLP and an enhanced sampling approach. The Neural Scene Representation (NSVF) [26] was introduced as a solution for efficient and high-fidelity rendering. To capture local characteristics within each voxel, NSVF employs a collection of implicit fields bounded by voxels, which are organized within a sparse voxel octree structure. This sparse voxel octree arrangement facilitates the rapid generation of new perspectives by disregarding voxels that do not contain relevant scene information. This method overcomes the limitations of traditional voxel-based rendering methods by modeling the underlying surface with a continuous signed distance function, which ensures high-quality rendering even for complex scenes. “bakes” NeRF [27] enables real-time rendering on commodity hardware. It reformulates NeRF’s architecture using sparse voxel grids with learned feature vectors, enabling efficient rendering without the need for original training data. EfficientNeRF [2] proposes effective sampling in both rough and fine stages to improve sampling efficiency. Additionally, the method incorporates a new caching mechanism that stores the entire scene, significantly accelerating rendering speed during testing. Moreover, to enhance model robustness during inference, randomization of camera position and direction is employed during training. These improvements, along with the introduction of a novel data structure for scene caching, contribute to a substantial increase in rendering speed and overall efficiency. MVSNeRF [28] utilizes MVSNet [29] to generate a feature volume for NeRF, enabling the synthesis of high-quality images with just 15 min of finetuning. However, the testing time for these methods still remains as long as that of the original NeRF.

### 2.4. Limitation of Existing Methods

Despite the significant progress made by these methods in view synthesis and NeRF training, they still have certain limitations. DS-NeRF [19] requires a set of 3D keypoints generated by COLMAP [30]. A prerequisite for the successful initialization of COLMAP is that there needs to be a sufficiently large translational movement of the relative poses between the photos, and the operation of DS-NeRF relies on COLMAP. This limitation prevents DS-NeRF from functioning properly in some scenarios. DONeRF [1] requires an additional depth oracle network during training, which could increase the computational time and make training more challenging. Most NeRF methods that use depth information only apply a depth loss to constrain the rendered depth values. In contrast, our approach starts from the principles of volume rendering and introduces a completely new way of generating rays, ensuring that the ray termination distribution aligns with the scene surface. This effectively reduces depth errors and allows for more accurate rendering of geometric information in the scene. EffiecientNeRF [2] requires scene caching during testing, which may not be feasible for large-scale scenes or devices with limited memory. Moreover, appropriate sampling is sensitive to the initial view direction, and training with a limited set of initial views may introduce bias towards those views. For surface rendering, the precise identification of an accurate surface is crucial to ensure consistent colors across different views. However, this task presents significant challenges that impede training convergence and result in undesirable blurring effects in the rendered images. In contrast, volume rendering methods require sampling a large number of points along the rays to accurately capture colors and achieve high-quality rendering. However, NeRF’s evaluation of each sampled point along the ray is inefficient. It takes approximately 22 s to use NeRF on an NVIDIA RTX 3060 to generate a 640 × 480 image.

Our key observation highlights the importance of restricting point sampling in empty spaces. By leveraging depth information, we can precisely pinpoint the surface location within the current scene and focus sampling near object surfaces. This approach substantially reduces the required quantity of sample points and enhances the training speed of NeRF. Additionally, depth information can effectively supervise NeRF, ensuring that the termination distribution of each light ray closely aligns with the actual object position.

## 3. Method

By integrating depth information into the traditional NeRF method, we achieved a termination distribution of rays that closely approximates the surface of the real scene. This effectively addresses the problem of shape-radiance ambiguity in traditional NeRFs.

**Figure 2:**
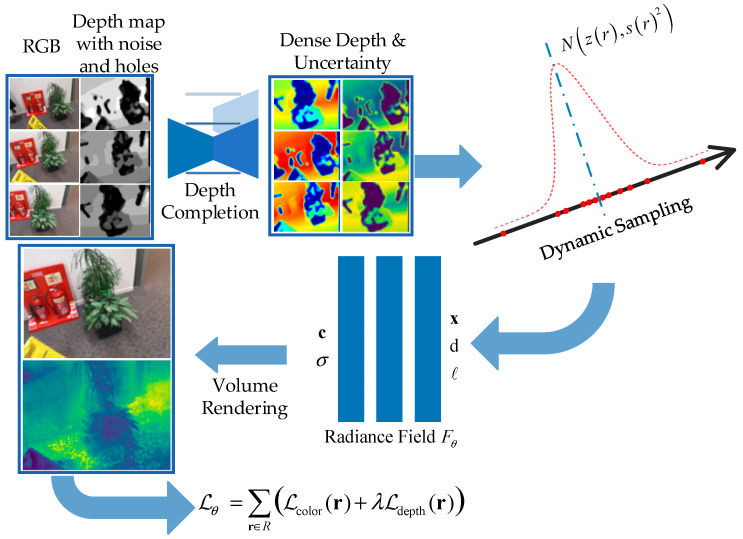
Overview of our radiance field optimization pipeline.

Our pipeline is shown in Figure 2. Given a set of RGB images and their corresponding depth maps, many of these depth maps have holes and noise. After depth completion, we obtain complete and accurate depth maps, along with uncertainty for each depth. Using this depth information, we guide the distribution of sampling points near the scene surfaces. Inputting the positions of these points and the camera viewpoint into the NeRF model yields the color and opacity of each point. Finally, through volume rendering, we generate the corresponding RGB images and depth maps.

We begin by revisiting the volumetric rendering technique, followed by an analysis of depth completion. Next, we analyze the location of sampling points guided by depth information. Finally, we conclude by discussing optimization with the depth constraint.

*a*. *Volume Rendering with Radiance Fields*

NeRF is a deep learning method that reconstructs 3D scenes from 2D images. It encodes a scene as a volume density and emitted radiance by employing a model called the neural radiance field, which represents density and color information within the 3D scene. This model comprises a feed-forward neural network that takes positions and orientations in 3D space as input and produces the corresponding density and color values at those positions. More specifically, for a given 3D point $x\in R^{3}$ and a specific direction d, NeRF provides a function to estimate the volume density σ and RGB color c:(σ,c)=f(x,d).

When NeRF renders an image with a given pose p, a series of rays is cast from the p’s center of projection o in the direction d. Since the rays have a fixed direction, the propagation distance can be parameterized by time: r(t)=o+td. We sample multiple points along each ray and input these sample points into the NeRF network, obtaining voxel density σ and radiance c for each sample point. By integrating the implicit radiance field along the ray, we estimate the color of the object surface from viewing direction d.

(1) $$\hat{C}(r)=\sum_{k=1}^{K}w_{k}c_{k}$$

(2) $$w_{k}=T_{k}\left(1-\exp(-\sigma_{k}\delta_{k})\right)$$

(3) $$T_{k}=\exp\left(-\sum_{k^{\prime }=1}^{k}\sigma_{k^{\prime }}\delta_{k^{\prime }}\right)$$

where $\delta_{k}=t_{k+1}-t_{k}$. $T_{k}$ is transmittance, which checks for occlusions by integrating the differential density between 1 to k. $w_{k}$ describes the contribution of each sampled point along the ray to the radiance. NeRF assumes that the scene exists within a range (1,k), and to ensure the sum of $w_{k}$ is equal to 1 a non-transparent wall is introduced at k. The final loss function of NeRF is as follows:

(4) $$L_{\mathrm{Color}}=\sum_{r\in R(p)}{\left\|\hat{C}(r)-C(r)\right\|}_{2}^{2}$$

where R(p) represents a series of rays emitted from a fixed pose, p.

*b*. *Depth Completion with Uncertainty*

Depth maps captured by RGB-D cameras in consumer devices often suffer from noise, holes, and missing regions. To address this issue, we employ depth completion algorithms to fill in the missing information and generate a complete depth image. We utilize the OpenCV-based depth completion method, which is designed to run efficiently on the CPU and provide real-time performance. The problem of depth completion can be described as follows:

Given an image $I\in {\mathbb{R}}^{M\times N}$, and a sparse depth map $D_{sparse}$, find $\hat{f}$ that approximates a true function $f:{\mathbb{R}}^{M\times N}\times {\mathbb{R}}^{M\times N}\to {\mathbb{R}}^{M\times N}$ where $f(I,D_{sparse})=D_{dense}$. The problem can be formulated as:

(5) $$min.||\hat{f}(I,D_{sparse})-f(I,D_{sparse})|{|}_{F}^{2}=0$$

Here, $D_{dense}$ is the output dense depth map with missing data replaced by their depth estimates.

Due to the input source originates from a LIDAR sensor and its suitability limited to large-scale outdoor scenes, we have made improvements to the IP_Basic [31] algorithm, tailoring it to better suit indoor scenarios, and it now supports RGB-D input. Specifically, we have implemented critical adjustments in the calculation methods for the near-plane mask and far-plane mask. These modifications have enabled our algorithm to effectively segment indoor scenes into distinct close-range and far-range areas. By accurately dividing the scene based on proximity, our enhanced algorithm delivers more precise and reliable results for indoor applications. The partitioning of these two areas significantly affects the accuracy of depth completion.

**Figure 3:**
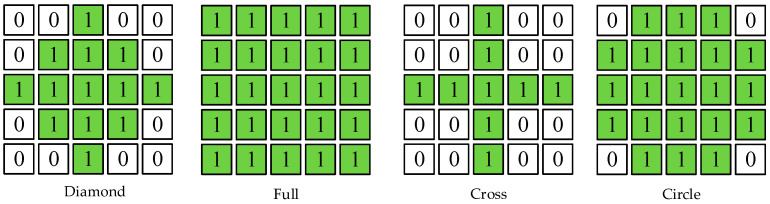
Using different kernel to process depth images.

The algorithm follows a series of steps to process the depth map effectively. First, we begin with preprocessing the depth map. Our dataset primarily consists of indoor scenes with depth values ranging from 0 to 20 m. However, some empty pixels have a value of 0, necessitating preprocessing before utilizing OpenCV operations. To address this issue, we invert the depths of valid (non-empty) pixels according to $D_{inverted}=D_{max}-D_{valid}$, creating a buffer zone between valid and empty pixels. In the subsequent steps, we employ OpenCV’s dilation operation to process the image. This operation can result in the blurring of nearby object edges. Another benefit of inverting the depth is to prevent such blurring. Next, we consider that the depth values of adjacent pixels in the depth map generally change smoothly unless near object edges. To tackle this, we start by filling empty pixels closest to valid pixels. We use OpenCV’s dilation operation, which involves sliding a kernel over the input image. At each kernel position, the dilate operation checks if at least one pixel under the kernel is non-zero. If it finds at least one non-zero pixel under the kernel, it sets the center pixel of the kernel to a non-zero value in the output image. The kernel’s design ensures that pixels with similar values are dilated to a common value. Figure 3 illustrates various potential shapes of the kernel. We assessed the effects of different kernel sizes and shapes, as shown in Table 1. Unlike IP_Basic [31], we found that a 4 × 4 kernel size produces the best results, and we use a 4 × 4 diamond kernel to dilate all valid pixels.

After the initial dilation operation, there may still be gaps in the depth map. We observe that adjacent sets of dilated depth values can be connected to establish object edges. To address this, we apply a 5 × 5 full kernel to close minor gaps in the depth map. Furthermore, to address any remaining gaps, we perform a dilation operation with a 7 × 7 kernel. This process selectively fills empty pixels while leaving the previously computed valid pixels unchanged. In the next step, we address larger gaps in the depth map that may not have been completely filled in the previous steps. Similar to the previous operations, we use a dilation operation with a 20 × 20 full kernel to fill any remaining empty pixels while ensuring that valid pixels remain unaltered. Following the preceding dilation operations, certain outliers may emerge. To eliminate these outliers, we employ a 5 × 5 kernel median blur. Subsequently, we use a 5 × 5 Gaussian blur to enhance the smoothness of local surfaces and soften the sharp edges of objects. Finally, the last step involves reverting back to the original depth according to $D_{output}=D_{max}-D_{inverted}$.

We evaluate the reliability of the depth estimation for each pixel. Let $d_{i}$ be the depth estimation for a pixel, and $d_{true,i}$ be the corresponding true depth value of the pixel. By taking the logarithm of the depth values, we can convert absolute errors into relative errors. In this way, whether the object is near or far, we can fairly evaluate the accuracy of the prediction. Then, the reliability of the depth for each pixel is calculated as follows:

(6) $$\sigma_{i}=|\ln(d_{i})-\ln(d_{\mathrm{true},i})|$$

**Figure 4:**
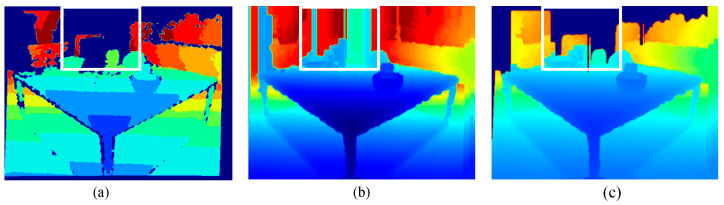
Comparison of depth completion effects. (**a**) Raw depth map with noise and holes. (**b**) IP_Basic (**c**) Modified IP_Basic.

Figure 4 clearly shows that our modified IP_Basic algorithm outperforms the original version in indoor scenes. The original algorithm considered tall objects like trees, utility poles, and buildings that extend beyond LIDAR points. It extrapolated the highest column values to the top of the image for a denser depth map, but this could lead to noticeable errors when applied in indoor settings. As seen in Figure 4b,c, our approach effectively rectifies errors in the depth values at the top of the depth map.

*c*. *Depth-Guided Sampling*

In 3D space, there are many empty (unoccupied) regions. Traditional NeRF methods tend to oversample these empty regions during training, resulting in significant computational overhead for NeRF. To tackle this problem, our proposed approach introduces a sampling strategy that leverages depth information to guide the sampling process effectively. For each pixel in each image of the training dataset, a ray is emitted. If the object surface is completely opaque, the ray will terminate at the object surface. We have access to the depth map corresponding to each frame, where each pixel represents the distance between the object and the camera in camera coordinates. We then perform sampling around this depth value. Specifically, each pixel $p_{i}$ is associated with a corresponding depth value $depth_{i}$ in the image $I\in {\mathbb{R}}^{M\times N}$. We denote the positions of the sampling points as points and ensure that they follow a Gaussian distribution with a mean of $depth_{i}$ and a standard deviation of $\sigma_{i}$. Therefore, the random variable points follows the Gaussian distribution:

(7) $$points\sim N(depth_{i},{\sigma_{i}}^{2})$$

The sampled points are strategically distributed around the depth, preventing excessive sampling in empty space and conserving computational resources. The sampling space is determined by the depth value of the object corresponding to the current pixel. Simply expanding the sampling range, as in the original NeRF approach, would inevitably result in inefficient sampling. This highlights one of the key reasons for the time-consuming nature of NeRF.

*d*. *Optimization with Depth Constrain*

To optimize the neural radiance field, the color $\hat{C}(r)$ of the object surface corresponding to each pixel is computed using Equation (1). In addition to the predicted color of a ray, the NeRF depth estimate $\hat{z}(r)$ and standard deviation $\hat{s}(r)$ are required to provide supervision to the radiance field based to the depth prior.

(8) $$\begin{array}{c} \hat{z}(r)=\sum_{k=1}^{K}w_{k}t_{k},\;\hat{s}{\left(r\right)}^{2}=\sum_{k=1}^{K}w_{k}{\left(t_{k}-\hat{z}(r)\right)}^{2} \end{array}$$

By minimizing the loss function $L_{\theta }$, we can obtain the optimal network parameter θ. $L_{\theta }$ consists of depth-related loss function $L_{\mathrm{depth}}$ and color-related loss function $L_{\mathrm{color}}$. Here, λ is a hyperparameter used to adjust the weights of $L_{\mathrm{depth}}$ and $L_{\mathrm{color}}$.

(9) $$L_{\theta }=\sum_{r\in R}(L_{\mathrm{color}}(r)+\lambda L_{\mathrm{depth}}(r))$$

The discrete forms of $L_{\mathrm{depth}}$ and $L_{\mathrm{color}}$ are given as follows:

(10) $$L_{\mathrm{Color}}=\sum_{r\in R(p)}{\left\|\hat{C}(r)-C(r)\right\|}_{2}^{2}$$

(11) $$L_{Depth}=\sum_{r\in R\left(p\right)}{\left\|\log\left(\hat{s}{\left(r\right)}^{2}\right)+\frac{{\left(\hat{z}(r)-z(r)\right)}^{2}}{\hat{s}{\left(r\right)}^{2}}\right\|}_{2}^{2}$$

By adopting this approach, NeRF is incentivized to terminate rays within a range close to the most confident surface observation based on the depth prior. Simultaneously, NeRF still retains a degree of flexibility in allocating density to effectively minimize color loss. Rays are cast from the camera coordinate center towards these 3D points, theoretically terminating at these 3D points. We apply a depth loss to encourage the termination distribution of the rays to be consistent with the 3D points in space.

## 4. Experiments

In this section, we compared our method to recently proposed approaches. Additionally, we conducted ablation experiments to verify the necessity of depth completion and depth-guided sampling. Finally, we compared the training speed of our algorithm under the same conditions with that of NeRF.

### 4.1. Experimental Setup

Our computational experiments were conducted on a system equipped with an Intel Core i9-13900K CPU, which has a 24-core architecture and a top clock speed of 5.8 GHz. The system also featured an NVIDIA GeForce RTX 4090 GPU, built on Ada Lovelace architecture, with 24 GB of GDDR6X memory and 16,384 CUDA cores.

We used the Adam optimizer with a learning rate of 6 × 10^−4^ to process rays in batches of 1024. The radiance fields were optimized for 50k iterations. To simulate a scenario involving the partial absence of depth information, we introduced random perturbations to the depth data in the dataset.

To quantitatively compare different view synthesis methods, we calculated the peak signal-to-noise ratio (PSNR), the Structural Similarity Index Measure (SSIM) [32], and the Learned Perceptual Image Patch Similarity (LPIPS) [33] on the RGB of new views. Moreover, we computed the root-mean-square error (RMSE) between the rendered depth maps generated by different methods and the depth information recorded by the sensor.

### 4.2. Basic Comparison

We compared our method with NeRF [3], Depth-Supervised NeRF [19], and NerfingMVS [18]. The results of the experiment in Table 2 demonstrate that our method achieves better performance than the baselines across all evaluation criteria.

**Figure 5:**
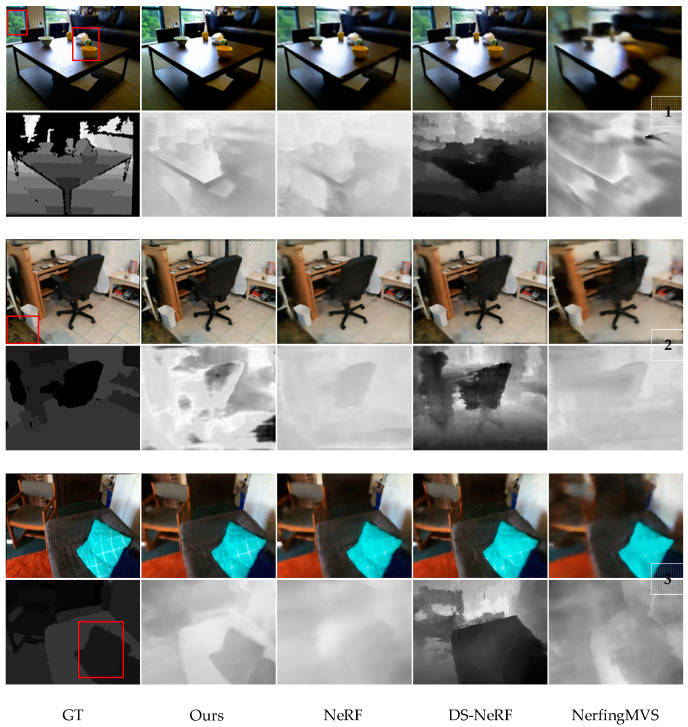
Comparison between rendered images and depths of the four test scenes and real images and depths.

Applying NeRF in scenarios with limited input views often leads to some artifacts. However, our proposed method addresses this issue by integrating dense depth priors accompanied by uncertainty. This incorporation leads to a remarkable reduction of artifacts compared to conventional baseline methods. Additionally, our approach achieves significantly enhanced accuracy in depth output and finer granularity in color representation. An illustrative example showcasing the effectiveness of our method can be observed in Example 1, as depicted in Figure 5. The images generated by our approach exhibit distinctly sharper edges for both the table and objects when contrasted with the output obtained using NeRF. The lack of depth supervision in NeRF poses considerable challenges in establishing a reliable correspondence between images, particularly when relying solely on RGB values. This challenge is further amplified in scenarios where the number of available images is limited, resulting in the proliferation of artifacts in the synthesized output.

Despite the limitations of sparse 3D point utilization [19], our method’s integration of dense depth priors and uncertainty estimation achieves remarkable results. It emerges as a highly promising solution for diverse scenarios. Our approach effectively mitigates artifacts, improves depth accuracy, and enhances color fidelity. Supervising depth information from both RGB data and 3D points enables us to establish reliable image correspondence and reduce artifacts. Our method outperforms traditional NeRF approaches, making it a promising solution for scenarios.

**Figure 6:**
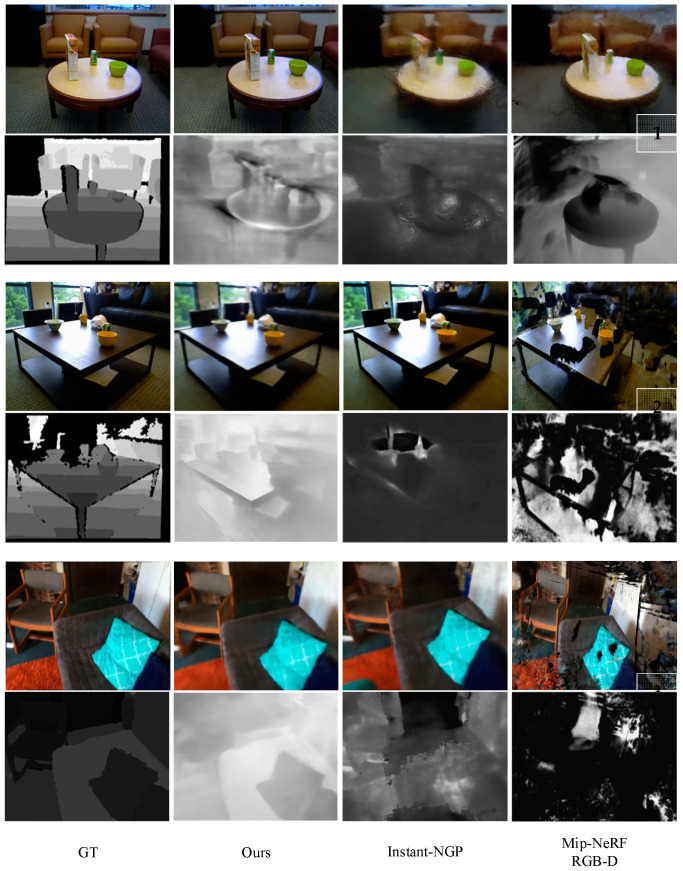
Comparing rendered images and depth data with real images and depth information from three test scenes.

In Figure 6, we compared our approach with two popular methods, Instant-NGP with depth supervision [34] and Mip-NeRF RGB-D [20]. Instant-NGP proposes a coding method that allows for the utilization of smaller-scale networks to implement NeRF without sacrificing accuracy. The network is enhanced using a multi-resolution hash table of feature vectors and optimized through stochastic gradient descent. The multi-resolution structure aids in GPU parallel computing and reduces computation by eliminating hash conflicts. Instant-NGP excels in capturing fine details, as demonstrated in Example 2, Figure 6, where it captures more high-frequency details. However, Instant-NGP comes with a higher requirement for the number of input views. In comparison to an equal number of input views, our method delivers superior overall performance, as illustrated in Example 3, Figure 6. Mip-NeRF RGB-D uses conical frustums instead of rays for volume rendering, allows one to account for the varying area of a pixel with distance from the camera center. In bounded environments, this design does not exhibit a clear advantage. In our method, we utilize a depth supervision mechanism, which enables us to concentrate exclusively on samples near the scene’s surface. This selective approach proves to be advantageous during the rendering phase, as we synthesize depth information for the new viewpoint by making use of the available depth information. By promoting the alignment of a ray’s termination distribution with the surface of the scene, we achieve a significant reduction in artifacts compared to the baseline methods. The outcome of this refinement process is evident in the form of a more precise depth output and a richer representation of colors, as explicitly demonstrated in Figure 6. These improvements are a result of our method’s ability to effectively handle outliers, its tailored training approach, and the selective utilization of depth information during the rendering process. Together, these advancements contribute to the production of high-quality rendered images with improved fidelity and visual appeal.

**Figure 7:**
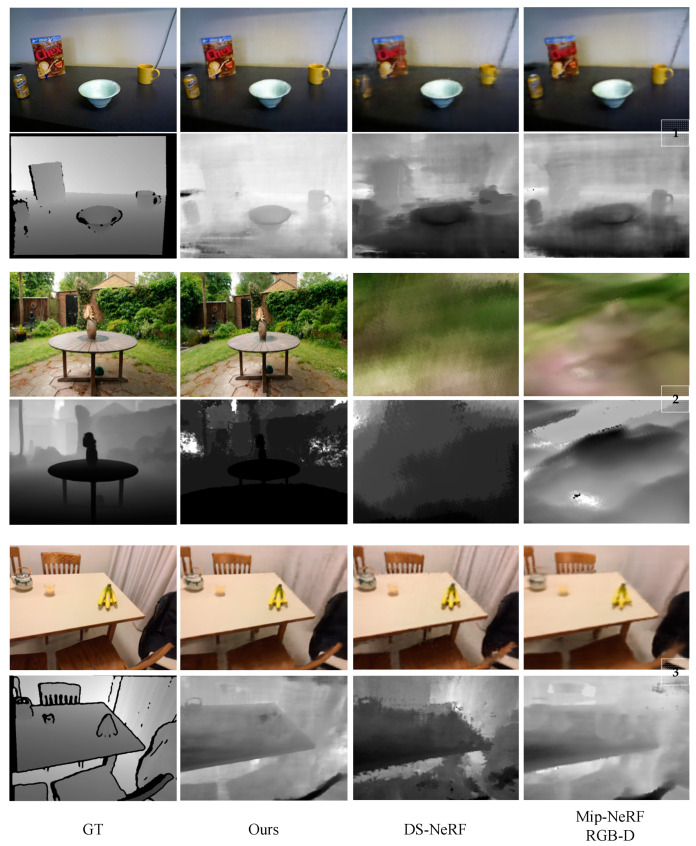
Basic comparison in real-world scenarios.

In order to further demonstrate the practicality of our method, we conducted experiments on real-world datasets. We selected three different scenarios and compared our method with two mainstream approaches: DS-NeRF and Mip-NeRF RGB-D. The results show that our method significantly outperforms the baseline algorithms. In Example 1 of Figure 7, the RGB image rendered by our method exhibits superior detail handling, particularly in the portion of the yellow cup. Simultaneously, the generated depth map also has clear contours. In Example 2 of Figure 7, the results of DS-NeRF and Mip-NeRF RGB-D are not satisfactory, but our method produces high-quality RGB images and depth maps. In Example 3 of Figure 7, although all the methods render fairly good RGB images, the quality of the depth map rendered by our method still exceeds the other methods.

Depth completion not only enables our method to generate higher quality depth maps, but also allows us to obtain the reliability of depth at each pixel during the depth completion process, providing valuable references for subsequent NeRF training. On the one hand, the reliability of pixel depth can determine the distribution of sampling points, thereby optimizing the rendering effect. On the other hand, when calculating depth loss, we can more reasonably handle points with lower reliability, thereby further improving the accuracy and stability of our method. These results show that even when working with real-world data, our approach, which relies on deep complementation and deep rays, can generate better quality results than other methods.

### 4.3. Ablation Study

**Figure 8:**
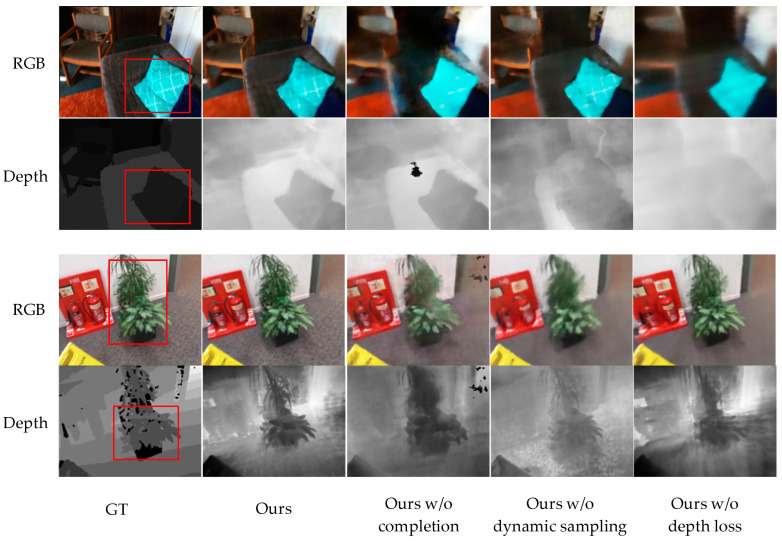
Rendered RGB and depth images for test views from ScanNet and 3DMatch.

To verify the impact of the added components, we performed ablation experiments on the ScanNet [35] and 3DMatch [36] datasets. The quantitative results (refer to Table 3) demonstrate that the complete version of our method achieves superior performance in terms of image quality and depth estimation. These findings are consistent with the qualitative results depicted in Figure 8.

**Without Completion** The exclusion of depth completion significantly impacts the accuracy of both depth estimation and color reproduction, primarily due to the presence of artifacts in areas lacking depth input. Moreover, even in regions characterized by low confidence in depth values, the outcomes exhibit inferior sharpness compared to those regions with higher confidence in depth values.

**Without Dynamic Sampling** In the absence of dynamic sampling guided by depth information, the termination depth of each ray may deviate from the actual scene conditions. By employing a dynamic sampling strategy that concentrates a majority of sampling points near the surface of the scene, the accuracy of depth is greatly enhanced. As depicted in Figure 8, significant improvements in RGB image quality are not observed after the addition of dynamic sampling. However, a notable enhancement has been observed in the quality of the depth map.

**Without Depth Loss** Lack of deep supervision leads to shape-radiance ambiguity. Despite the generation of high-quality RGB images from new perspectives, an accurate representation of the scene’s geometry remains unattainable. Merely a fraction of the generated depth map provides accurate depth information, as the differences in depth across the remaining areas are negligible, rendering it inadequate for portraying the actual scene depth.

### 4.4. Training Speed

**Figure 9:**
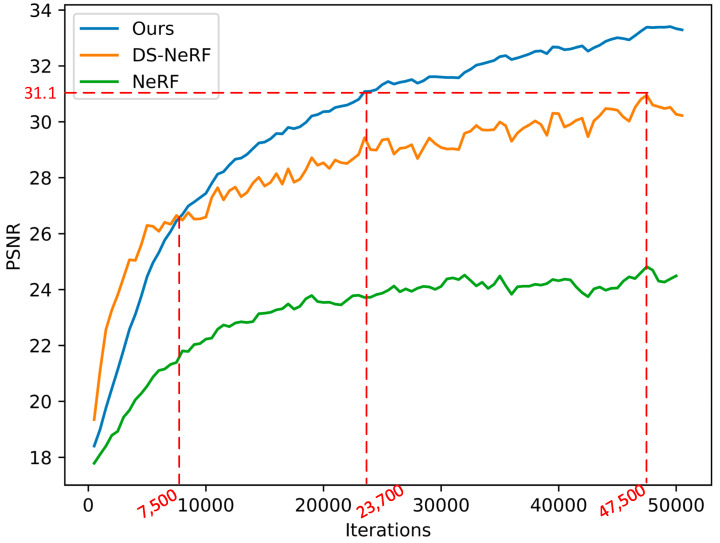
Training speed comparison.

To quantify the speed improvements in NeRF training, We conducted a comprehensive comparison and analysis of the training speeds of Depth-NeRF, NeRF, and DS-NeRF under appropriate settings. The evaluation of view synthesis quality on training views was performed using PSNR, considering varying numbers of input views obtained from the 3DMatch dataset [36]. To assess the training speed performance, we plotted the PSNR values on training views against the number of training iterations, as shown in Figure 9.

We trained Depth-NeRF, DS-NeRF, and NeRF models under the same training conditions, and the results indicate that Depth-NeRF achieves a peak PSNR quality comparable to that of NeRF and DS-NeRF, but with considerably fewer iterations. From Figure 9, it can be observed that between 0 and 7500 iterations, DS-NeRF exhibits higher PSNR values compared to our approach. However, beyond the 7500 iteration mark, our method begins to outperform DS-NeRF in PSNR performance. Noteworthy is the fact that our method achieves DS-NeRF’s maximum PSNR after just 24,000 iterations, showcasing a remarkable 51% reduction in iteration count compared to DS-NeRF. It can be observed that the training speed and rendering quality of the two methods incorporating depth supervision are significantly better than those of the original NeRF. Due to our utilization of a mechanism that dynamically distributes ray-sampling points guided by depth information, our training speed is even faster than DS-NeRF, while ultimately achieving higher PSNR. This implies superior rendering quality.

## 5. Conclusions

In this study, we proposed a novel view synthesis method that utilizes neural radiance fields and dense depth priors. Our method utilizes depth completion on scene depth maps to provide dense and accurate depth information for NeRF training and efficient sample collection. This allows for improved rendering quality and more accurate, continuous representations of scene depth. We demonstrate that our dense depth information with uncertainty effectively guide the NeRF optimization, resulting in considerably improved image quality for novel views and more precise depth estimates compared to alternative approaches. Furthermore, our approach exhibits a notable reduction of 18% in the training time required by NeRF.

Although our approach demonstrates advantages across various datasets, there is still the potential for further enhancement. The current positional encoding method has room for improvement, especially in representing high-frequency details, and future research can focus on finding more suitable positional encoding approaches. Additionally, our method has a relatively high demand for GPU memory, which may pose challenges in specific application scenarios. To reduce memory requirements, future work can concentrate on optimizing the network structure of NeRF. Overall, we perceive our method as a meaningful progression towards making NeRF reconstructions more accessible and applicable in common settings.

## Figures and Tables

**Table 1:** Impact of dilation kernel size and shape on depth completion.

Kernel Size	RMSE (mm)	MAE (mm)
3 × 3	1649.97	367.06
**4 × 4**	**1427.74**	**335.67**
5 × 5	1545.85	349.45
Full	1545.85	349.45
Circle	1528.45	342.49
Cross	1521.95	333.94
**Diamond**	**1512.18**	**333.67**

**Table 2:** Quantitative comparison of three representative scenes from ScanNet and 3DMatch.

	3DMatch Rgbd-Scenes-v2-Scene_01				ScanNet Scene0002_00				3DMatch 7-Scenes-Pumpkin			
	PSNR↑	SSIM↑	LPIPS↓	Depth-RMSE↓	PSNR↑	SSIM↑	LPIPS↓	Depth-RMSE↓	PSNR↑	SSIM↑	LPIPS↓	Depth-RMSE↓
NeRF	29.44	0.613	**0.046**	0.485	30.03	0.527	0.190	**0.092**	30.11	0.691	0.122	0.198
DS-NeRF	31.55	0.854	0.080	1.113	30.02	0.535	0.208	1.347	30.21	0.810	0.075	1.124
NerfingMVS	29.39	0.537	0.274	1.098	29.23	**0.546**	0.266	0.989	29.62	0.759	0.094	1.163
Mip-NeRF RGB-D	28.740	0.198	0.332	0.846	28.18	0.219	0.436	0.723	28.42	0.798	0.070	0.542
Instant-NGP	30.12	0.768	0.196	0.727	29.84	0.482	0.367	0.535	30.73	**0.887**	0.182	0.194
Depth-NeRF	**32.47**	**0.883**	0.077	**0.462**	**30.10**	0.534	**0.186**	0.116	**31.66**	0.836	**0.067**	**0.151**

**Table 3:** Quantitative comparison of two representative scenes from ScanNet and 3Dmatch.

	3DMatch 7-Scenes-Fire				ScanNet Scene0002_00			
	PSNR↑	SSIM↑	LPIPS↓	Depth-RMSE↓	PSNR↑	SSIM↑	LPIPS↓	Depth-RMSE↓
Depth-NeRF w/o completion	29.78	0.549	0.038	0.173	28.55	0.496	0.278	0.204
Depth-NeRF w/o depth loss	30.31	0.572	0.099	1.115	29.59	0.534	0.320	0.524
Depth-NeRF w/o dynamic sampling	30.40	0.564	0.128	0.498	29.48	0.531	0.231	0.489
Depth-NeRF	**30.41**	**0.592**	**0.026**	**0.152**	**30.03**	**0.566**	**0.186**	**0.201**

## Data Availability

The source code used during the current study is available from https://github.com/Goodyenough/Depth-NeRF (accessed on 15 March 2024).
